# Key Vitamin D Target Genes with Functions in the Immune System

**DOI:** 10.3390/nu12041140

**Published:** 2020-04-19

**Authors:** Oona Koivisto, Andrea Hanel, Carsten Carlberg

**Affiliations:** School of Medicine, Institute of Biomedicine, University of Eastern Finland, FI-70211 Kuopio, Finland; oonkoi@student.uef.fi (O.K.); andrea.hanel@uef.fi (A.H.)

**Keywords:** Vitamin D, VDR, epigenome, transcriptome, gene regulation, vitamin D target genes, immune system, monocytes, PBMCs

## Abstract

The biologically active form of vitamin D_3_, 1α,25-dihydroxyvitamin D_3_ (1,25(OH)_2_D_3_), modulates innate and adaptive immunity via genes regulated by the transcription factor vitamin D receptor (VDR). In order to identify the key vitamin D target genes involved in these processes, transcriptome-wide datasets were compared, which were obtained from a human monocytic cell line (THP-1) and peripheral blood mononuclear cells (PBMCs) treated in vitro by 1,25(OH)_2_D_3_, filtered using different approaches, as well as from PBMCs of individuals supplemented with a vitamin D_3_ bolus. The led to the genes *ACVRL1*, *CAMP*, *CD14*, *CD93*, *CEBPB*, *FN1*, *MAPK13*, *NINJ1*, *LILRB4*, *LRRC25*, *SEMA6B*, *SRGN*, *THBD*, *THEMIS2* and *TREM1*. Public epigenome- and transcriptome-wide data from THP-1 cells were used to characterize these genes based on the level of their VDR-driven enhancers as well as the level of the dynamics of their mRNA production. Both types of datasets allowed the categorization of the vitamin D target genes into three groups according to their role in (i) acute response to infection, (ii) infection in general and (iii) autoimmunity. In conclusion, 15 genes were identified as major mediators of the action of vitamin D in innate and adaptive immunity and their individual functions are explained based on different gene regulatory scenarios.

## 1. Introduction

Vitamin D_3_ is known as a micronutrient that is essential for calcium homeostasis and bone formation [1,2]. However, vitamin D_3_ is also a pre-hormone that is endogenously produced when skin is exposed to sufficient amounts of UV-B [3]. Vitamin D_3_ affects gene regulation after it is converted via hydroxylation at carbon 25 (providing 25-hydroxyvitamin D_3_) and at carbon 1 to 1α,25-dihydroxyvitamin D_3_ (1,25(OH)_2_D_3_), which is the high affinity ligand of the transcription factor vitamin D receptor (VDR) [4]. Thus, ligand-activated VDR binds to accessible genomic sites in the vicinity of its target genes and modulates their transcription [5].

Interestingly, a fully potent VDR protein evolved some 550 million years ago in a boneless vertebrate, i.e., at a time when there was no need for calcium homeostasis and bone formation [6,7,8]. VDR’s first evolutionary function was the control of metabolism, in order to support the evolving immune system of ancestral vertebrates with energy [9]. Thus, VDR and its ligand first specialized in the modulation of innate and adaptive immunity, such as fighting against bacterial and viral infections [10,11] and preventing autoimmune diseases, such as multiple sclerosis and rheumatoid arthritis [12,13], before they took on the additional task of regulating bone metabolism. Thus, vitamin D deficiency is causing an increase in bone disease, such as rickets [14], and it may also be one of the reasons for increased vulnerability, particularly in elderly persons, against viral infections, such as the recent coronavirus (COVID-19) outbreak [15].

Since monocytes are the most vitamin D responsive cell type in the immune system, the human monocytic leukemia cell line THP-1 is often used as a model system for the description of vitamin D signaling [16]. Peripheral blood mononuclear cells (PBMCs), which are a mixture of lymphocytes and monocytes isolated from blood, are an interesting and easily accessible primary vitamin D-responsive tissue [17]. In this study, we will use data from both cellular systems, in order to analyze the regulation of vitamin D target genes in innate and adaptive immunity.

The key regulatory regions of a gene are its transcription start site (TSS) and enhancer(s). The latter are stretches of genomic DNA that bind one or several signal-responsive transcription factors [18]. Single enhancers have a dominant transcription factor binding site, while super-enhancers are formed by multiple single enhancers [19]. Genomic DNA is always embedded in chromatin, the major function of which is to control the access of transcription factors to enhancer and TSS regions [20,21]. Accordingly, the epigenome of a cell is represented by covalent and structural modifications of its chromatin [22]. For example, active chromatin is detected via acetylated histone H3 proteins at position lysine 27 (H3K27ac) [23], while tri-methylated histone H3 protein at position lysine 4 (H3K4me3) indicates active TSS regions [24].

The next-generation sequencing method, chromatin immunoprecipitation followed by sequencing (ChIP-seq) is used for the genome-wide detection of transcription factor binding and histone modifications [25], while formaldehyde-assisted identification of regulatory elements followed by sequencing (FAIRE-seq) determines genome-wide chromatin accessibility [26]. Interestingly, a number of attributes of the epigenome are vitamin D sensitive, such as the binding of transcription factors like VDR and pioneer factors, and the accessibility of chromatin and changes in histone modifications [4]. A VDR ChIP-seq meta-analysis indicated that there are on average more than 10,000 VDR binding loci per cell type [27]. However, only a few hundred of these sites are persistently occupied by VDR and some 2000 are bound transiently, while the majority of them are only found at later points in time [28]. Based on the chromatin model of vitamin D signaling [4], the looping of these VDR-bound enhancers to TSS regions modulates the activity of hundreds of vitamin D target genes in VDR expressing tissues. These vitamin D-induced shifts in the epigenome and transcriptome represent the molecular basis of vitamin D signaling [5]. 

In this study, we aimed to provide a shortlist of vitamin D target genes that are of key importance in the immune system. On the basis of the published genome-wide data for THP-1 cells, we analyzed the epigenome profile of these genes and the dynamics of their transcriptional response to stimulation with 1,25(OH)_2_D_3_. This allowed the categorization of the target genes into groups representing different physiological functions of vitamin D in the context of immunity.

## 2. Material and Methods

The study involved three main steps, which are described below.

Inspection of vitamin D target gene lists: three different RNA-seq datasets were used [29,30,31], which had been analyzed by the algorithm DESeq2 [32] for differentially expressed genes. Moreover, the THP-1 dataset had been filtered by the machine learning method, self-organizing map (SOM) [28] or for the 100 genes showing either the highest basal activity, inducibility (fold change (FC)) or significance (lowest *p*-value) [33]. The in vitro treated PBMCs [31] were used either unfiltered or filtered for the top 100 genes with regard to basal activity, FC or *p*-value. The in vivo PBMC data were not further filtered. Venn diagrams were created by using the webtool jvenn [34] (http://jvenn.toulouse.inra.fr/app/index.html).

Epigenomic characterization of enhancers and TSS regions: published epigenome-wide data for THP-1 cells were used, which had been treated for 24 h with 10 nM 1,25(OH)_2_D_3_ or solvent (0.1% EtOH): ChIP-seq data of VDR [28], H3K27ac [35] and H3K4me3 [35] as well as FAIRE-seq data [29]. Using the IGV-browser [36] all VDR-bound enhancers 1 Mb up- and downstream of the genes’ TSSs as well as the TSS regions themselves were first visually inspected for VDR binding, markers of active chromatin and accessible chromatin. Then, VDR classification [28] and statistically significant (*p* < 0.05) responsiveness to 1,25(OH)_2_D_3_ treatment of enhancers and TSS regions [28,29,35] was checked based on data provided by the respective publications. When the criteria for a super-enhancer were not fulfilled, i.e., three VDR binding sites within 20 kb, one of which needs to be a strong persistent or transient site and a continuous H3K27ac mark throughout its whole enhancer [33], the closest single enhancer to the TSS carrying a strong persistent or transient VDR site was selected. The epigenome profile of TSS regions was also inspected for statistically significant (*p* < 0.05) responsiveness to 1,25(OH)_2_D_3_ treatment. Where necessary, higher magnification was used in order to distinguish enhancers close to the TSS from direct TSS binding.

Analysis of time-dependent gene expression: published RNA-seq data of a time course (2.5, 4 and 24 h) of 1,25(OH)_2_D_3_ stimulation in THP-1 cells [29] were used, in order to categorize the genes as primary (statistically significant (*p* < 0.05) increase in expression within 4 h) or secondary vitamin D targets. The genes were categorized into tertials (top, mid and low) with respect to the basal activity (expression level of solvent control), inducibility (FC at 24 h) and sensitivity (*p*-value at 24 h). The classification of the genes is based on the steepness of their expression curves, which mostly correlates with their inducibility (group 1: steepest curves, group 2: intermediate increase, group 3: no major increase after 4 h).

## 3. Results

### 3.1. Selection of Key Immune-Related Genes

**Figure 1:**
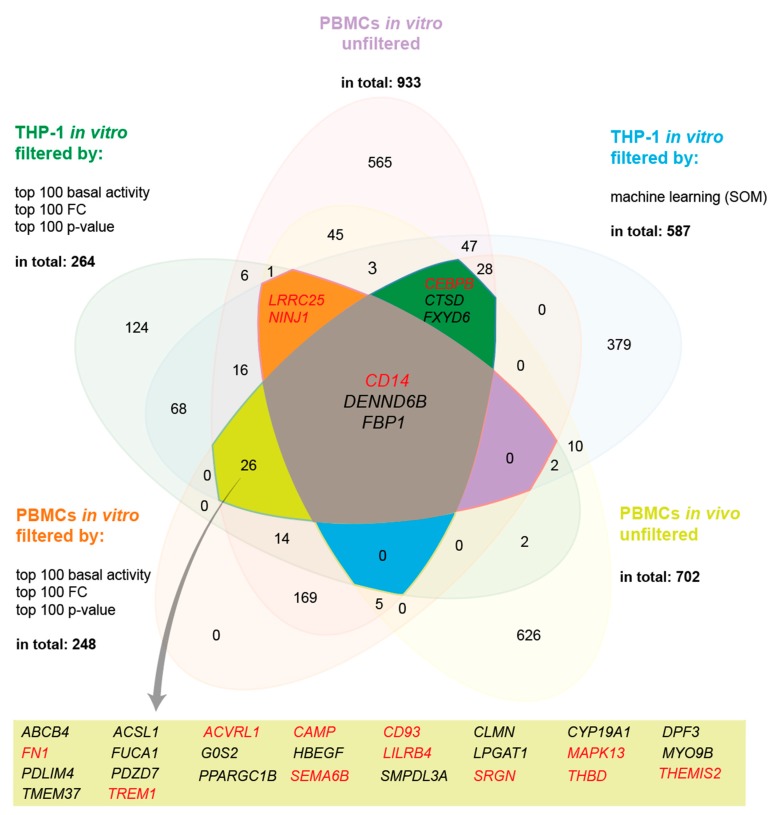
Key vitamin D target genes in immune-related cell types. A Venn diagram represents vitamin D target genes that are common in THP-1 cells stimulated in vitro in three biological repeats for 24 h with 1,25(OH)_2_D_3_; PBMCs of 12 individuals treated in vitro in a single repeat for 24 h with 1,25(OH)_2_D_3;_ and PBMCs of five individuals supplemented once for 24 h with a vitamin D_3_ bolus. Identification of differentially expressed genes was performed in all three datasets by DESeq2, but in vitro treated THP-1 and PBMCs were each filtered by two different approaches. Thirty-four genes were chosen for further inspection, 3 of which were found in all five datasets (center) and further 31 genes overlapped in four of the five lists. Immune-related genes are highlighted in red.

In order to identify common vitamin D target genes in immune related cell types, we used the results of three different RNA-seq datasets. These were from THP-1 cells, which had been treated in three biological repeats for 24 h with 10 nM 1,25(OH)_2_D_3_ [29]; PBMCs of 12 healthy individuals, which were stimulated in vitro in a single repeat for 24 h with 10 nM 1,25(OH)_2_D_3_ [31]; and PBMCs of five healthy individuals, which had been supplemented once for 24 h with a vitamin D_3_ bolus of 2000 µg [30]. The THP-1 dataset was filtered by the machine learning method self-organizing map (SOM) [28] or for the top 100 genes with respect to basal activity, FC or *p*-value [33], which provided 587 and 264 target genes, respectively (Figure 1). Similarly, in vitro treated, unfiltered PBMCs showed 933 target genes or 248 genes after filtering for the top 100 genes with respect to basal activity, FC or *p*-value. In vivo PBMCs reported 702 vitamin D target genes. A Venn diagram of these five gene lists highlighted the genes *CD14* (CD14 molecule), *DENND6B* (DENN domain containing 6B) and *FBP1* (fructose-bisphosphatase 1) as common targets. However, under the condition that only four of five datasets need to overlap, a further 31 genes were identified.

The known functions of all 34 genes were inspected via information collected at GeneCards (www.genecards.org). Eight genes were dismissed from the list because their main functions are closely related to metabolism, 10 further genes were excluded since no relation to the immune system was identified, and one was omitted due to the fact that no information on its function was available. This resulted in 15 genes: *ACVRL1* (activin A receptor like type 1), *CAMP* (cathelicidin antimicrobial peptide), *CD14*, *CD93* (CD93 molecule), *CEBPB* (CCAAT enhancer binding protein beta), *FN1* (fibronectin 1), *MAPK13* (mitogen-activated protein kinase 13), *NINJ1* (ninjurin 1), *LILRB4* (leukocyte immunoglobulin like receptor B4), *LRRC25* (leucine rich repeat containing 25), *SEMA6B* (semaphorin 6B), *SRGN* (serglycin), *THBD* (thrombomodulin), *THEMIS2* (thymocyte selection associated family member 2) and *TREM1* (triggering receptor expressed on myeloid cells 1) (Figure 1). Since immune and cancer cells share the property of rapid growth [37], they also overlap in their list of vitamin D target genes related to rapid cellular growth. Therefore, we had to carefully evaluate which proliferation-related gene to select. Thus, the *MAPK13* gene was included, while the *G0S2* (G0/G1 switch 2) gene was left out, since no publication has indicated that it has a direct link to immune cells.

In summary, five lists of vitamin D target genes, which were obtained from two cell types (THP-1 and PBMCs), two types of treatment (1,25(OH)_2_D_3_ in vitro or vitamin D_3_ bolus in vivo) and three ways of filtering (SOM, top 100 for basal activity, FC and *p*-value as well as unfiltered), led to a list of 34 genes, 15 of which are related to immune function.

### 3.2. Epigenomic Profile of 15 Key Target Genes

Using published data for THP-1 cells, the epigenomic profile of the 15 selected vitamin D target genes was inspected 1 Mb up- and downstream of the TSS for 1,25(OH)_2_D_3_-dependent binding of VDR [28], histone markers of active chromatin (H3K27ac) [35] and active TSS regions (H3K4me3) [35] as well as accessible chromatin [29]. The genes were categorized into four classes based on whether VDR bound to super-enhancers or a single enhancer and whether these enhancers showed a statistically significant (*p* < 0.05) response to 1,25(OH)_2_D_3_ treatment on the level of at least one of the four epigenome-wide datasets (Table 1).

**Figure 2:**
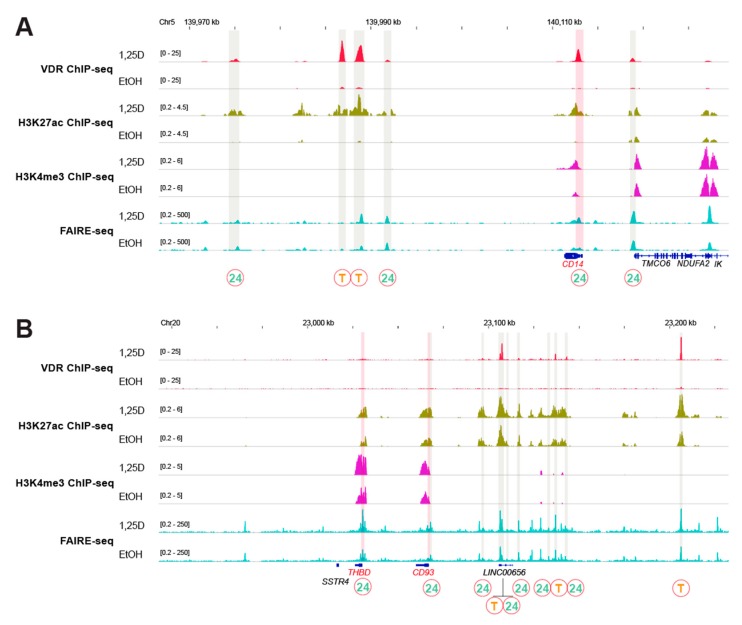
Vitamin D target genes regulated by super-enhancers. The IGV browser was used to visualize VDR ChIP-seq results (red) obtained in THP-1 cells that had been treated for 24 h with 1,25(OH)_2_D_3_ (1,25D) or solvent (EtOH) [28]. VDR-bound enhancers are shaded in grey and ligand-dependent TSS regions of vitamin D target genes in red. The type of VDR binding sites at enhancers is indicated as persistent (P), transient (T) and 24 h only (24) [28]. These data are compared with ChIP-seq results obtained under the same conditions for histone markers of active chromatin (H3K27ac, green) [35] and active TSS regions (H3K4me3, purple) [35] as well as with FAIRE-seq data (turquoise) [29]. The peak tracks display merged data from the three biological repeats. Gene structures are shown in blue and the vitamin D target genes *CD14* (**A**) and *THBD/CD93* (**B**) are highlighted in red. The genomic regions 1 Mb up- and downstream of the gene’s TSS were inspected but only the areas relevant for 1,25(OH)_2_D_3_-dependent regulation are displayed.

**Figure A1:**
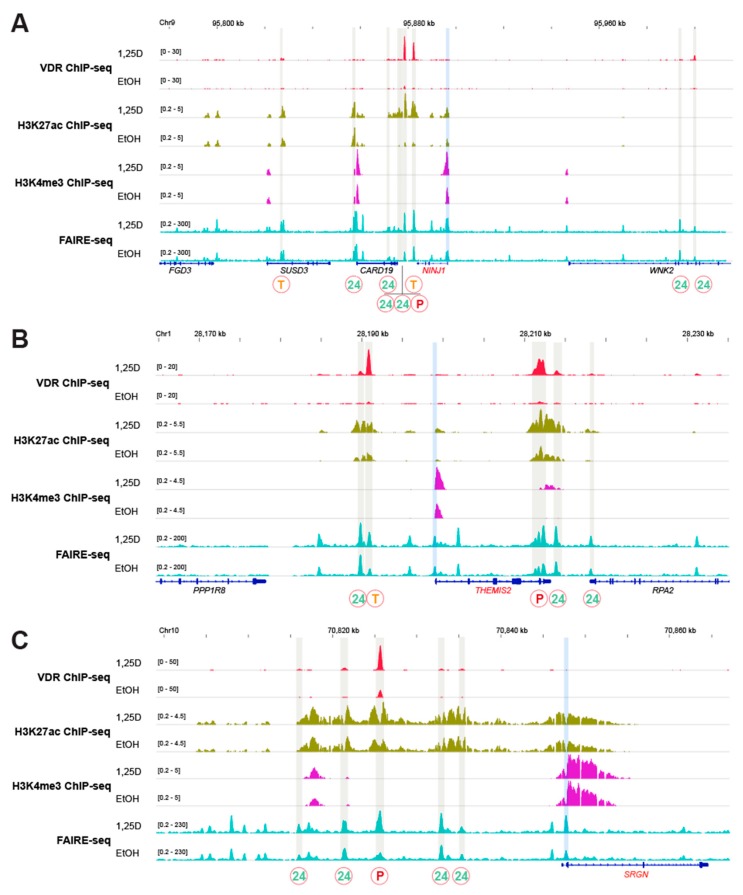
Vitamin D target genes controlled by super-enhancers. The IGV browser was used to visualize VDR ChIP-seq results (red) obtained in THP-1 cells that had been treated for 24 h with 1,25(OH)_2_D_3_ (1,25D) or solvent (EtOH) [28]. VDR-bound enhancers are shaded in grey and TSS regions of vitamin D target genes in blue. The type of VDR binding sites at enhancers is indicated as persistent (P), transient (T) or 24 h only (24) [28]. These data are compared with ChIP-seq results obtained under the same conditions for histone markers of active chromatin (H3K27ac, green) [35] and active TSS regions (H3K4me3, purple) [35] as well as with FAIRE-seq data (turquoise) [29]. The peak tracks display merged data from the three biological repeats. Gene structures are shown in blue and the vitamin D target genes *NINJ1* (**A**), *THEMIS2* (**B**) and *SRGN* (**C**) are highlighted in red. The genomic regions 1 Mb up- and downstream of the gene’s TSS were inspected but only the areas relevant for 1,25(OH)_2_D_3_-dependent regulation are displayed.

The *CD14* gene (Figure 2A) is a master example of the first epigenome group being regulated by a ligand-dependent super-enhancer (T-T-24), which is located some 25 kb downstream of the TSS. The two strong transient VDR binding sites as well as the H3K27ac signal of active chromatin throughout the whole super-enhancer are significantly (*p* < 0.05) affected by ligand. Moreover, 1,25(OH)_2_D_3_-dependent binding of VDR as well as of H3K27ac and H3K4me3 markers were detected at the TSS region of the gene. The genes *NINJ1* (Figure A1A) and *THEMIS2* (Figure A1B) are additional members of this epigenome group with ligand-dependent super-enhancers (P-T-24 and P-24-24) but non-ligand-dependent TSS regions.

The second epigenomic group contains the genes *CD93* and *THBD*, which reside next to each other and share the same non-ligand responsive super-enhancer (T-24-24) (Figure 2B). This applies also to the super-enhancer of the *SRGN* gene (P-24-24) (Figure A1C), which is the third member of this epigenome group. The TSS regions of the genes *CD93* and *THBD* respond to 1,25(OH)_2_D_3_ but not that of the *SRGN* gene.

**Figure 3:**
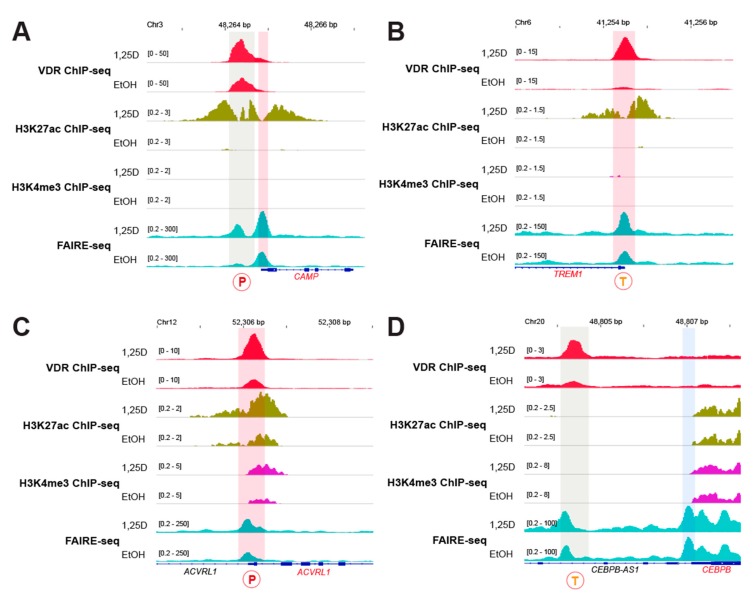
Vitamin D target genes regulated by single enhancers. The IGV browser was used to visualize VDR ChIP-seq results (red) obtained in THP-1 cells that had been treated for 24 h with 1,25(OH)_2_D_3_ (1,25D) or solvent (EtOH) [28]. VDR-bound enhancers are shaded in grey, ligand-dependent TSS regions in red and non-ligand-dependent TSS regions in blue. The type of VDR binding sites at enhancers is indicated as persistent (P) or transient (T) [28]. These data are compared with ChIP-seq results obtained under the same conditions for histone markers of active chromatin (H3K27ac, green) [35] and active TSS regions (H3K4me3, purple) [35] as well as with FAIRE-seq data (turquoise) [29]. The peak tracks display merged data from the three biological repeats. Gene structures are shown in blue and the vitamin D target genes *CAMP* (**A**), *TREM1* (**B**), *ACVRL1* (**C**) and *CEBPB* (**D**) are highlighted in red.

**Figure A2:**
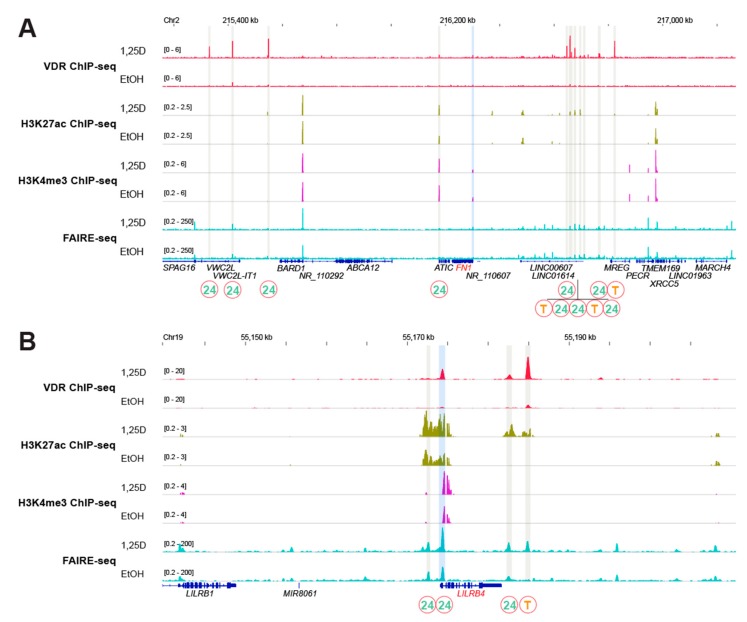
Vitamin D target genes controlled by ligand-dependent single enhancers. The IGV browser was used to visualize VDR ChIP-seq results (red) obtained in THP-1 cells that had been treated for 24 h with 1,25(OH)_2_D_3_ (1,25D) or solvent (EtOH) [28]. VDR-bound enhancers are shaded in grey and non-ligand-dependent TSS regions in blue. The type of VDR binding sites at enhancers is indicated as transient (T) or 24 h only (24) [28]. These data are compared with ChIP-seq results obtained under the same conditions for histone markers of active chromatin (H3K27ac, green) [35] and active TSS regions (H3K4me3, purple) [35] as well as with FAIRE-seq data (turquoise) [29]. The peak tracks display merged data from the three biological repeats. Gene structures are shown in blue and the vitamin D target genes *FN1* (**A**) and *LILRB4* (**B**) are highlighted in red. The genomic regions 1 Mb up- and downstream of the gene’s TSS were inspected but only the areas relevant for 1,25(OH)_2_D_3_-dependent regulation are displayed.

**Figure A3:**
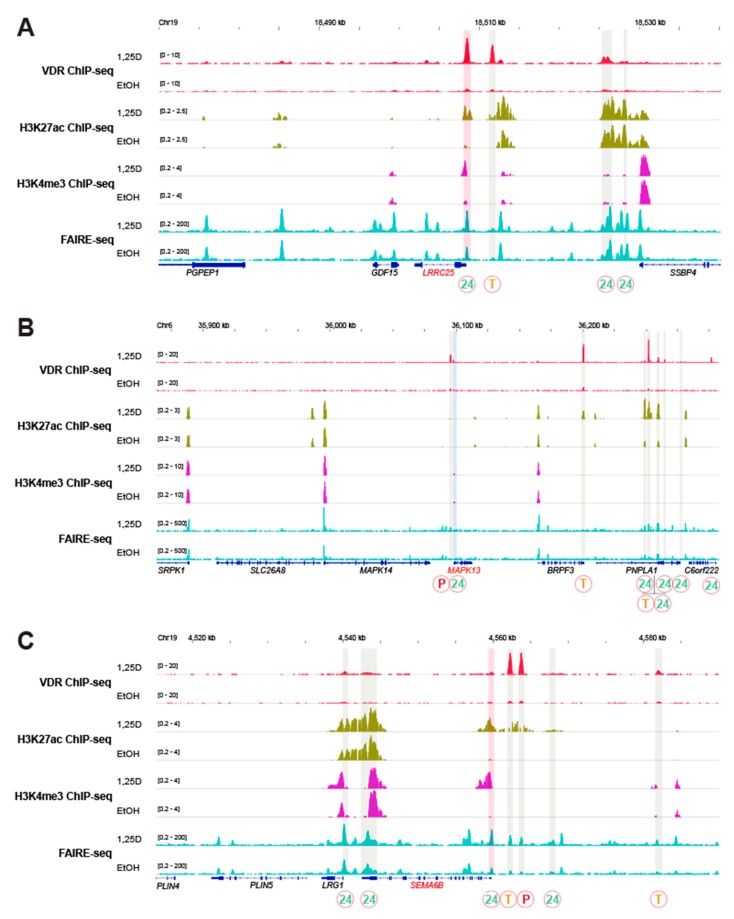
Vitamin D target genes controlled by non-ligand-dependent single enhancers. The IGV browser was used to visualize VDR ChIP-seq results (red) obtained in THP-1 cells that had been treated for 24 h with 1,25(OH)_2_D_3_ (1,25D) or solvent (EtOH) [28]. VDR-bound enhancers are shaded in grey, ligand-dependent TSS regions in red and non-ligand-dependent TSS regions in blue. The type of VDR binding sites at enhancers is indicated as persistent (P), transient (T) and 24 h only (24) [28]. These data are compared with ChIP-seq results obtained under the same conditions for histone markers of active chromatin (H3K27ac, green) [35] and active TSS regions (H3K4me3, purple) [35] as well as with FAIRE-seq data (turquoise) [29]. The peak tracks display merged data from the three biological repeats. Gene structures are shown in blue and the vitamin D target genes *LRRC25* (**A**), *MAPK13* (**B**) and *SEMA6B* (**C**) are highlighted in red. The genomic regions 1 Mb up- and downstream of the gene’s TSS were inspected but only the areas relevant for 1,25(OH)_2_D_3_-dependent regulation are displayed.

The nine remaining key immune-related genes are regulated by single enhancers carrying either a persistent or transient VDR site. The third epigenomic group is formed by the genes *CAMP* (Figure 3A), *FN1* (Figure A2A), *LILRB4* (Figure A2C) and *TREM1* (Figure 3B) being regulated by ligand-dependent enhancers, which are within the TSS region in the case of *CAMP* and *TREM1*. The TSS regions of the genes *CAMP* and *TREM1* are ligand-dependent but not that of the genes, *LILRB4* and *FN1*. The genes *ACVRL1* (Figure 3C), *CEBPB* (Figure 3D), *LRRC25* (Figure A3A), *MAPK13* (Figure A3B) and *SEMA6B* (Figure A3C) form the fourth epigenomic group, since they carry non-ligand responsive single enhancers. Interestingly, compared with the majority of the other genes, the distance of these enhancers to the TSS region is short (4 kb or less). The TSS regions of the genes *ACVRL1*, *LRRC25* and *SEMA6B* are sensitive to 1,25(OH)_2_D_3_ but not those of *CEBPB* and *MAPK13*.

Taken together, the most characteristic property of vitamin D target genes is its VDR-bound enhancers [38]. Accordingly, the epigenomic profile of the 15 key genes is primarily distinguished by the presence of a super-enhancer versus a single enhancer and its response to 1,25(OH)_2_D_3_. 

### 3.3. Dynamic Response of the Transcriptome

**Figure 4:**
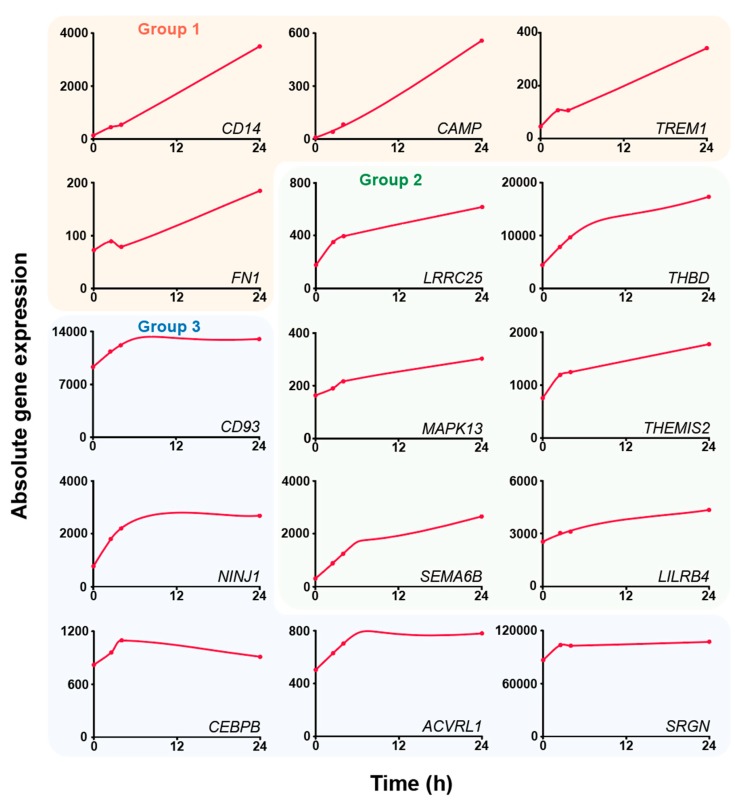
Dynamic response of gene expression to 1,25(OH)_2_D_3_. Based on published RNA-seq data from THP-1 cells [29], the increase in expression after 1,25(OH)_2_D_3_ stimulation of the 15 key genes is displayed for basal activity (solvent control) and the time points 2.5, 4 and 24 h. Genes are classified into three groups based on the steepness of the expression curves.

The dynamic response of the 15 key immune-related genes to vitamin D was analyzed based on the published RNA-seq time course data from THP-1 cells, which had been stimulated for 2.5, 4 and 24 h with 1,25(OH)_2_D_3_ [29] (Figure 4). With the exception of the genes *FN1* and *CEBPB,* all other genes are primary vitamin D targets (Table 2). The curve of the expression time course of the genes *CD14*, *CAMP*, *TREM1* and *FN1* showed the steepest increase, which defined group 1. The genes *LRRC25*, *THBD*, *MAPK13*, *THEMIS1*, *SEMA6B* and *LILRB* present an intermediate response to 1,25(OH)_2_D_3_ and formed group 2. In contrast, the genes *CD93*, *NINJ1*, *CEBPB*, *ACVRL1* and *SRGN* of group 3 do not show any significant increase after 4 h, i.e., a more or less horizontal curve. The members of group 1 are characterized by a low basal activity and high inducibility (Table 2). Interestingly, all four genes in this group are regulated by ligand-inducible enhancers (Table 1). In contrast, the genes in group 3 have mid to high basal activity but low inducibility and their enhancers are not vitamin D sensitive. Finally, the genes in group 2 have basal activity and inducibility in the mid-range.

In summary, the time-dependent gene expression profile of the 15 key genes allows their categorization into three groups, that is, they display high, mid and low responsiveness to vitamin D stimulation. Interestingly, highly responsive genes carry ligand-sensitive enhancers, while the enhancers of low responsive genes do not show a reaction to 1,25(OH)_2_D_3_ stimulation.

## 4. Discussion

**Figure 5:**
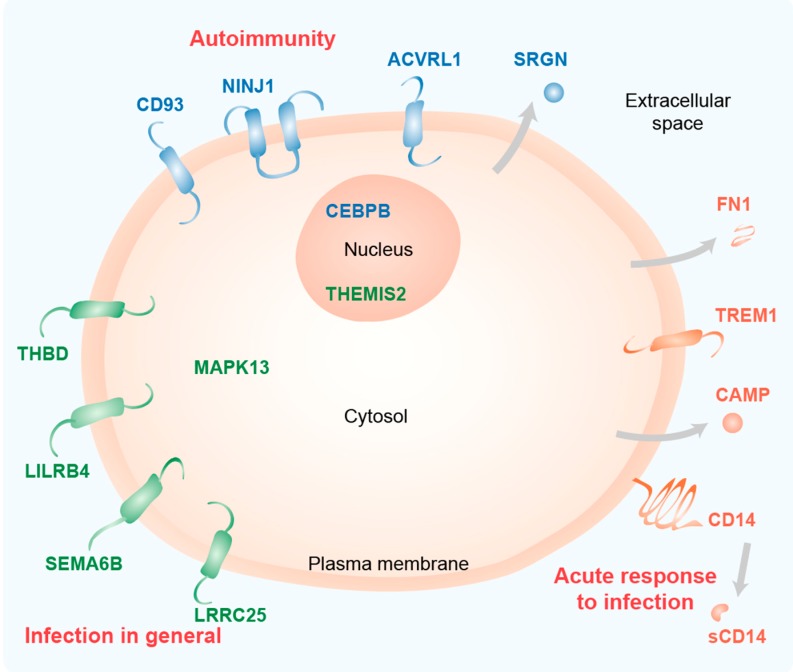
Functional profile of key immune-related vitamin D target genes. Schematic picture of a cell indicating the main location of the proteins encoded by the 15 key genes. The information is based on GeneCards (www.genecards.org) and publications cited in the text. The classification of the proteins (group 1: orange, group 2: green, group 3: blue) is based on their transcriptome profile (Figure 4). The main immune-related function of the protein groups is indicated in red.

This study uses data from vitamin D-stimulated monocytes and PBMCs to highlight a shortlist of 15 genes that are the major targets of vitamin D within the human immune system. As expected for immune-related genes, most of the proteins encoded by these genes are located in or at the plasma membrane (ACVRL1, CD14, CD93, LILRB4, LRRC25, NINJ1, SEMA6B, THBD, TREM1) or are even secreted (CAMP, FN1 and SRGN) (Figure 5). Furthermore, the transcription factor CEBPB and the Toll-like receptor (TLR) signaling scaffold protein THEMIS2 act in the nucleus and the kinase MAPK13 acts in the cytoplasm.

The majorly increased production of the anti-microbial peptide CAMP is a well-known example of the action of vitamin D in supporting innate immunity in the fight against bacteria, such as *Mycobacterium tuberculosis* [39,40]. The glycoprotein CD14 shows highest expression in monocytes and macrophages. It is anchored via glycosylphosphatidylinositol on the surface of the plasma membrane and is also found in a secreted form (sCD14). CD14 acts as a co-receptor for the pattern recognition receptors TLR1-4, 6, 7 and 9 [41]. It delivers the pathogen-associated molecule lipopolysaccharide (LPS), which is produced exclusively by gram-negative bacteria, to TLR4 resulting in pro-inflammatory responses [42]. The transmembrane glycoprotein TREM1, which is found in monocytes, macrophages and neutrophils, is also heavily involved in TLR4 signaling, i.e., it promotes inflammation in response to bacterial infection [43]. TREM1 is already known as a vitamin D target gene [44]. Similarly, the gene encoding for the extracellular matrix protein FN1 has long been known as a vitamin D target [45]. The protein is secreted by a large variety of cell types, such as macrophages, fibroblasts and epithelial cells. FN1 functions in cell adhesion and wound healing but also participates in LPS/TLR4 signaling, i.e., in the inflammatory responses [46]. Thus, the proteins encoded by the highly vitamin D-responsive genes of group 1 are all involved in acute responses to infection.

THBD is a transmembrane protein expressed in endothelial cells, monocytes and macrophages [47]. The traditional role of THBD is to bind thrombin and turn its pro-coagulative action to anti-coagulative, i.e., reducing blood clots. However, THBD can also bind LPS and induces its binding to the complex of CD14 and TLR4, i.e., it prevents the pro-inflammatory consequences of NF-ĸB signaling [48]. The THBD gene is known as a key vitamin D target both in monocytes [49] and in PBMCs [50]. LILRB4 is an inhibitory immuno-regulatory receptor that acts on antigen-presenting cells like dendritic cells, macrophages, monocytes and microglia. It affects TNF production and bactericidal activity [51] as well as the modulation of the differentiation of regulatory T cells [52]. The LILRB4 gene is known as a vitamin D target gene in PBMCs [53]. The transmembrane protein SEMA6B belongs to a the semaphorin protein family, members of which have immune functions related to the control of cell movements and cell-cell communication [54]. Interestingly, the family member SEMA3B is known as a vitamin D target gene in bone [55]. The transmembrane protein LRRC25 is found in monocytes, dendritic cells, granulocytes and T lymphocytes. It acts as a negative regulator of the signaling pathways of NF-ĸB [56] and interferon [57], i.e., it suppresses the production of inflammatory cytokines and modulates the response to viral infections. The latter is a long overlooked effect of vitamin D on the immune system [58,59] and a reason why vitamin D deficiency may lead to high vulnerability against viral infections in the elderly, but also in school children [15,60,61].

The kinase MAPK13 is involved in LPS/TLR4 signaling and together with MAPK11 it regulates cytokine-induced inflammatory responses [62]. The *MAPK13* gene is expressed in a large variety of cell types and it has already been described as a vitamin D target in skeletal muscle [63]. The TLR signal transduction modulatory protein THEMIS2 is expressed in B lymphocytes, macrophages and dendritic cells and regulates LPS-induced TNF production downstream of TLR4 [64]. Moreover, the protein is involved in the development of T lymphocytes and serves as a marker of monocytic differentiation [65]. Thus, the proteins encoded by the mid vitamin D responsive genes of group 2 are primarily involved in general responses to infection.

CD93 is a transmembrane glycoprotein that is expressed primarily in endothelial cells but also in granulocytes, monocytes, platelets and stem cells [66]. The protein is involved in several processes of innate immunity, such as adhesion, phagocytosis and inflammation [67]. In the context of the latter, CD93 may act as a plasma membrane receptor for DNA for delivery to endosomal TLR9. Moreover, CD93 boosts the inflammatory response of monocytes by increasing LPS recognition by TLR4. CD93 may have a protective role in autoimmune encephalomyelitis via the control of the severity of inflammation, apoptosis and bystander neuronal injury [68]. NINJ1 is a transmembrane protein expressed primarily in myeloid and endothelial cells [69] but it was first described in the peripheral nervous system inducing neurite extension. In the latter context, NINJ1 was found to be involved in the immune pathogenesis of multiple sclerosis [70]. NINJ1 functions in cell adhesion and inflammation, such as leukocyte migration to sites of inflammation in the endothelium. CEBPB is a transcription factor that plays diverse roles in inflammation, e.g., through T helper cell 17 (T_H_17)-dependent regulation of inflammation in models of multiple sclerosis [71]. The *CEBPB* gene has already been described as a vitamin D target in myeloid leukemia cells [72]. ACVRL1 is a transmembrane protein of the transforming growth factor beta superfamily, which mediates the bone morphogenetic protein (BMP) 9- and BMP10-induced signaling that orchestrates the development of blood vessels [73]. This relates to the control of monocyte to macrophage differentiation [74]. SRGN is a secreted proteoglycan of endothelial cells, monocytes, mast cells and lymphocytes [75]. LPS up-regulates *SRGN* expression in macrophages, suggesting the protein plays a role of in storage and secretion of inflammatory mediators [76]. Thus, the proteins encoded by the low vitamin D responsive genes of group 3 are primarily involved in autoimmunity.

## 5. Conclusions

Transcriptome group 1 (*CAMP*, *CD14*, *FN1*, *TREM1*) encodes for proteins that relate to acute response to infection, in particular to the LPS/TLR4 signaling pathway. The genes show low basal expression but highest inducibility. Accordingly, their enhancers are all ligand-dependent. Transcriptome group 2 (*LILRB4*, *LRRC25*, *MAPK13*, *SEMA6B*, *THBD*, *THEMIS2*) is characterized by intermediate responsiveness on the transcriptome and epigenome level. These genes encode for proteins having a general function in infection. Transcriptome group 3 (*ACVRL1*, *CD93*, *CEBPB*, *NINJ1*, *SRGN*) encodes preferentially for proteins related to autoimmunity. The genes are characterized by high basal activity and low inducibility. Correspondingly, the enhancers of the genes are not ligand responsive. Thus, the physiological need for fast reactions to external threats had selected for genes with ligand-responsive enhancers, while genes involved in long-term responses, such as in autoimmunity, do not require a ligand responsive enhancer.

Taken together, we suggest that the highlighted 15 genes are the most relevant targets of vitamin D in the context of immunity and may be selected as biomarkers in clinical practice for the personalized diagnosis of the connection between vitamin D deficiency and immune-related diseases.

## Figures and Tables

**Table 1:** Epigenomic classification of vitamin D target genes based on super-enhancers or single enhancers and their responsiveness to 1,25(OH)_2_D_3_. The types of vitamin D receptor (VDR) binding sites at enhancers are indicated as persistent (P), transient (T) and 24 h only (24) [28].

Gene Symbol	Distance Enhancer-TSS (kb)	Enhancer Constellation	Super-Enhancer	1,25(OH)_2_D_3_-Dependency
*CD14*	25	T-T-24	yes	yes
*NINJ1*	20	P-T-24	yes	yes
*THEMIS2*	19	P-24-24	yes	yes
*CD93*	40	T-24-24	yes	no
*SRGN*	32	P-24-24	yes	no
*THBD*	80	T-24-24	yes	no
*CAMP*	0.5	P	no	yes
*FN1*	359	T	no	yes
*LILRB4*	10	T	no	yes
*TREM1*	0	T	no	yes
*ACVRL1*	0	P	no	no
*CEBPB*	2.5	T	no	no
*LRRC25*	4	T	no	no
*MAPK13*	3.5	P	no	no
*SEMA6B*	3	T	no	no

**Table 2:** Transcriptomic profile of key immune-related vitamin D target genes in THP-1 cells. Based on published RNA-seq data of a time course (2.5, 4 and 24 h) of 1,25(OH)_2_D_3_ stimulation in THP-1 cells [29], the 15 key genes were categorized as primary or secondary vitamin D targets and into tertials (top, mid and low) with respect to their basal activity (expression level with solvent control), their inducibility (fold change at 24 h) and sensitivity (*p*-value at 24 h). The classification of the genes is based on the steepness of the dynamic response of gene expression to 1,25(OH)_2_D_3_ stimulation (Figure 4).

Gene Symbol	Primary Target Gene?	Basal Activity	Fold Change (24 h)	*p*-Value (24 h)	Transcriptome Group
*CAMP*	yes	low	top	low	1
*CD14*	yes	low	top	top	1
*FN1*	no	low	mid	mid	1
*TREM1*	yes	low	top	top	1
*LILRB4*	yes	top	low	mid	2
*LRRC25*	yes	mid	top	top	2
*MAPK13*	yes	low	mid	low	2
*SEMA6B*	yes	mid	top	mid	2
*THBD*	yes	top	mid	top	2
*THEMIS2*	yes	mid	mid	mid	2
*ACVRL1*	yes	mid	low	mid	3
*CD93*	yes	top	low	low	3
*CEBPB*	no	top	low	low	3
*NINJ1*	yes	mid	mid	top	3
*SRGN*	yes	top	low	low	3
